# Prognostic Role of Monocytic Myeloid-Derived Suppressor Cells in Advanced Non-Small-Cell Lung Cancer: Relation to Different Hematologic Indices

**DOI:** 10.1155/2021/3241150

**Published:** 2021-10-11

**Authors:** Asmaa M. Zahran, Helal F. Hetta, Zeinab Albadry M. Zahran, Alaa Rashad, Amal Rayan, Dalia O. Mohamed, Zeinab Ahmed Abd Elhameed, Salah M. Khallaf, Gaber El-Saber Batiha, Yasir Waheed, Khalid Muhammad, Hanaa Nafady-Hego

**Affiliations:** ^1^Clinical Pathology Department, South Egypt Cancer Institute, Assiut University, Egypt; ^2^Department of Medical Microbiology and Immunology, Faculty of Medicine, Assiut University, Assiut 71526, Egypt; ^3^Department of Clinical Pathology, Faculty of Medicine, Assiut University, Assiut, Egypt; ^4^Department of Chest Diseases and Tuberculosis, Qena Faculty of Medicine, South Valley University, Egypt; ^5^Clinical Oncology Department, Faculty of Medicine, Assiut University, Egypt; ^6^Radiation Oncology Department, South Egypt Cancer Institute, Assiut University, Egypt; ^7^Medical Oncology Department, South Egypt Cancer Institute, Assiut University, Egypt; ^8^Department of Pharmacology and Toxicology, Faculty of Veterinary Medicine, Damanhour University, Damanhour City, Egypt; ^9^Foundation University Medical College, Foundation University Islamabad, Islamabad 44000, Pakistan; ^10^Department of Biology, College of Science, United Arab Emirates University, Al Ain 15551, UAE

## Abstract

**Methods:**

We recruited 40 cases of advanced NSCLC, stages III and IV, aged > 18–<70 years old, and eligible to receive chemotherapy with or without radiotherapy, along with 20 healthy controls of comparable age and sex; after diagnosis and staging of patients, blood samples were collected for flow cytometric detection of Mo-MDSCs.

**Results:**

Significant accumulation of Mo-MDSCs in patients compared to their controls (*p* < 0.0001). Furthermore, these cells accumulated significantly in stage IV compared to stage III (*p* = 0.006) and correlated negatively with overall survival (*r* = −0.471, *p* = 0.002), lymphocyte to monocyte ratio (*r* = −0.446, *p* = 0.004), and mean platelet volume to platelet count ratio (MPV/PC) (*r* = −0.464, *p* = 0.003), patients with Mo‐MDSCs < 13% had significantly better survival than those with Mo‐MDSCs ≥ 13% (*p* = 0.041).

**Conclusion:**

Mo-MDSCs represent one of the key mechanisms in the immunosuppressive tumor microenvironment (TME) to play major roles not only in the carcinogenesis of lung cancer but also in disease progression and prognosis and, in addition, predict the efficacy of immune checkpoint inhibitors; our results provided some support to target Mo-MDSCs and needed to be augmented by further studies.

## 1. Introduction

Globally, lung cancer is one of the leading causes of cancer-related death [1–3]. Although non-small-cell lung cancer (NSCLC) immunotherapy has fortunately emerged as a relatively promising area of research, immune checkpoint inhibitors have found an influential lantern for NSCLC patients. However, much work remains to elucidate lung tumor immunobiology and how alternative tumor microenvironments (TME) can affect patient survival across different NSCLC subtypes [4].

Recently, studies focused on TME and its role in tumor resistance; tumor suppressor cells within TME, namely, myeloid-derived suppressor cells (MDSCs), greatly attenuate the tumor response to chemotherapy and regrettably immunotherapy and subsequently affect NSCLC prognosis [5].

MDSCs encompass a range of immature cells whose unifying characteristics are their myeloid origin and ability to suppress T cell activation and T cell function. Phenotypically, these cells are defined by several markers; none of them is characteristic of MDSCs; the CD11b marker is expressed by all MDSCs [6]; there are two major subtypes of MDSCs; monocytic MDSCs express CD14, and polymorphonuclear MDSCs express CD15 and CD66b; both types express CD33 in addition to CD11b with the absence of HLA-DR.

Growth factors controlling myelopoiesis could induce the accumulation and augment the suppressive activity of MDSCs, including GM-CSF and G-CSF in cancer patients [7]. C/EBP*β* transcription factor, which is known to control emergency myelopoiesis, is expressed in chronic inflammation in many solid tumors and different inflammatory conditions, including infection, autoimmunity, obesity, and stress. These conditions have led to the hypothesis that chronic inflammation is a mechanism that increases the risk of cancer and tumor progression by acting as a driving force for MDSCs and subsequently suppressing antitumor immunity [8].

Vascular endothelial growth factor (VEGF), upregulated by hypoxia-inducible factor-1 in hypoxic TME, supports tumor progression through neovascularization. Studies done in NSCLC explored that VEGF attracts MDSCs to the tumor site and further promotes tumor progression [9].

Potent proinflammatory mediators such as IL-6, IL-1*β*, IL-17, and TNF-*α* accumulate in TME of many cancers to participate in tumor induction and progression; in addition, they induce overexpression and augmented suppressive activity of MDSCs [10–12]; other mediators including prostaglandin E2, cyclooxygenase 2, and proinflammatory calcium-binding proteins S100A8 and S100A9 are implicated in increasing immunosuppressive activity of MDSCs [13, 14].

MDSCs suppress both innate and adaptive immunity through cell to cell contact with their components, with T cells through sequestering the essential amino acids, cysteine, important for T cell activation. Furthermore, they downregulate the production of macrophage production of IL-12 favoring the development of tumor-promoting macrophage phenotype. In addition, they inhibit NK-mediated tumor cell lysis and recruit Tregs into the tumor site [15, 16]. Moreover, MDSCs downregulate L-selectin on circulating naïve T cells, therefore suppressing T cell activation [17].

Additionally, MDSCs may contribute to carcinogenesis and tumor progression through nonimmunosuppressive mechanisms. Immature myeloid cells directly contribute to skin tumor development by recruiting IL-17-producing CD4+ T cells [18]. In addition, MDSCs endow stem-like quality to breast cancer cells through IL6/STAT3 and NO/NOTCH cross-talk signaling [19]. Moreover, MDSCs could enhance the stemness of cancer cells by inducing microRNA101 and suppressing the corepressor gene C-terminal-binding protein-2 [20].

Huang et al. [21] reported that Mo-MDSCs significantly increased in the peripheral blood of patients with NSCLC compared to healthy controls and correlated with worse prognosis. The current study is aimed at providing the predictive and prognostic role of Mo-MDSCs in advanced NSCLC relating them to different hematologic indices.

## 2. Patients and Methods

This study was a case-controlled study carried out at South Egypt Cancer Institute and Assiut University Hospital and approved by the ethical committee of Assiut University (approval ID no. 17300417). Informed consent was taken from all study participants. The study was conducted in accordance with the Declaration of Helsinki. All experiments were performed in accordance with relevant guidelines and regulations.

We recruited 40 cases of advanced NSCLC, stages III and IV, aged > 18–<70 years old, and eligible to receive chemotherapy with or without radiotherapy, along with 20 healthy controls of comparable age and sex. Informed consent in written form was taken from all participants. The study objectives were explained to the participants, and then, blood samples were collected by sterilized and safe maneuvers.

We excluded patients with early stages, pretreated patients with chemotherapy, and patients with concurrent excruciating infection.

After diagnosis and staging of patients and before the start of any line of treatment, blood samples were collected for flow cytometric detection of Mo-MDSCs. Systemic chemotherapy was the treatment commonly received in the form of platinum doublets (carboplatin or cisplatin plus either pemetrexed, paclitaxel, gemcitabine, or vinorelbine); some patients especially those with ECOG-PS3 received single agent chemotherapy, while in patients with stage III, concurrent chemoradiation with a 3-dimensional conformal radiotherapy was received after induction chemotherapy.

Lymphocyte to monocyte ratio (LMR) was calculated by dividing the absolute lymphocytic count by the absolute monocytic count of the peripheral blood.

Mean platelet volume to platelet count ratio (MPV/PC) was calculated by dividing the mean platelet volume by total platelet count in *μ*l of peripheral blood.

### 2.1. Flow Cytometric Detection of Mo-Myeloid Derived Suppressor Cells (Mo-MDSCs)

**Figure 1:**
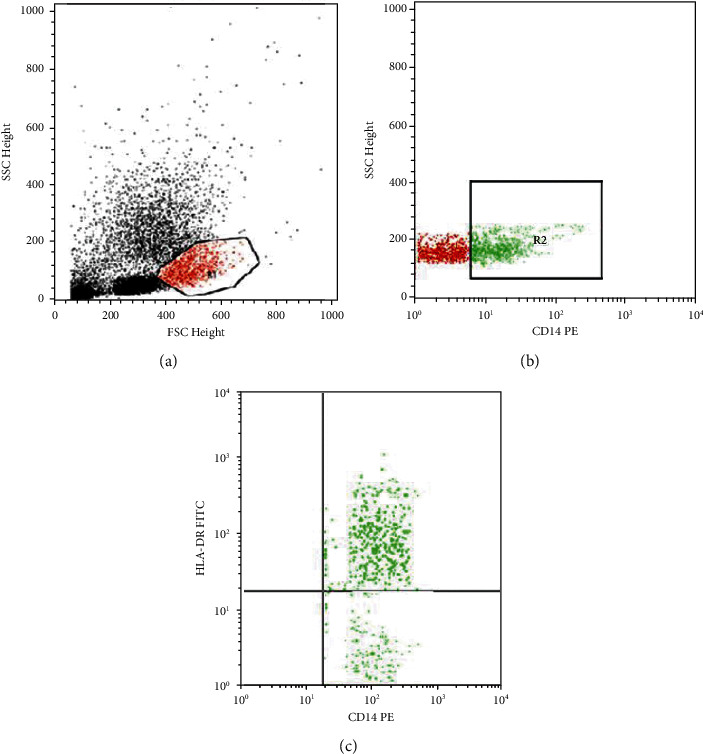
Flow cytometric detection of Mo-myeloid derived suppressor cells (Mo-MDSCs). (a) Forward and side scatter histogram was used to define the monocyte population (R1). (b) The expression of CD14 was assessed in monocytes population (R1) and CD14+ cells were then gated. (c) Then, the expression of HLA-DR on CD14+ cells (R2) was detected, to define CD14+HLA-DR- cells (Mo-MDSCs). Mo-MDSCs were expressed as a percentage of CD14+ cells.

To determine Mo-MDSCs, 100 *μ*l of blood sample was stained with 10 *μ*l of FITC-conjugated CD14 and 10 *μ*l of Per-CP-conjugated anti-HLA-DR; both were purchased from Becton Dickinson Biosciences, USA. Analysis was done by FACSCalibur flow cytometry with Cell Quest software (Becton Dickinson Biosciences, USA). Twenty thousand events were analyzed, and an isotype-matched negative control was used for each sample. The gating strategies to detect the percentage of CD14+HLA-DR- cells (Mo-MDSCs) are shown in Figure 1.

### 2.2. Statistics

The Shapiro-Wilk test was used to detect normality of our data; all data were normally distributed except percentage monocytes, Mo-MDSCs, absolute monocytic count, absolute lymphocytic count, MPV/PC ratio, and age with *p* values < 0.001, <0.001, 0.002, 0.04, 0.036, and 0.021, respectively, for whom nonparametric tests were applied.

Descriptive statistics including percentages, mean, median, and standard error were used and inferential statistics to determine the significance of data, including independent sample *t*-test, Mann–Whitney *U* test, Kruskal-Wallis test, and chi-square test. ROC curve was used to find a cutoff value for Mo-MDSCs, Spearman rho correlation was used to determine the degree of association between scale variables, and all data were analyzed using SPSS version 26 and considered significant at *p* value < 0.05. Overall survival (OS) was calculated from the time of diagnosis to time of death or last follow-up recorded in patients' files.

## 3. Results

### 3.1. Accumulation of Mo-MDSCs in NSCLC Patients

At first, our results elucidated a significant accumulation of Mo-MDSCs in NSCLC patients compared with their comparable healthy controls (*p* < 0.0001) (Table 1).

The mean age of the study patients was 59.5 years with male to female ratio of 1.7 : 1; although smoking was established as a risk factor for lung cancer, 72.5% of the study patients were either never smokers or past smokers. ECOG-PS is one of the two most commonly used performance scales; considering that NSCLC is a debilitating disease and commonly manifested at a later stage, poor performance status was evident in our study (70% of the patients had ECOG‐PS > 1); adenocarcinoma was the commonest pathologic type expressed in 57.5% of the patients, and as expected, stage IV was evident in 42.5% of the patients; the rest of the characteristics are illustrated in Table 2.

As expected, the significant accumulation of Mo-MDSCs in stage IV than stage III confirms the possible role of these cells in disease progression and indirectly referred to the role of immune-mediated destruction of tumor cells; however, these cells did not exhibit any significant change with other clinical characteristics (Table 3).

### 3.2. Correlations between Mo-MDSCs and Overall Survival

**Figure 2:**
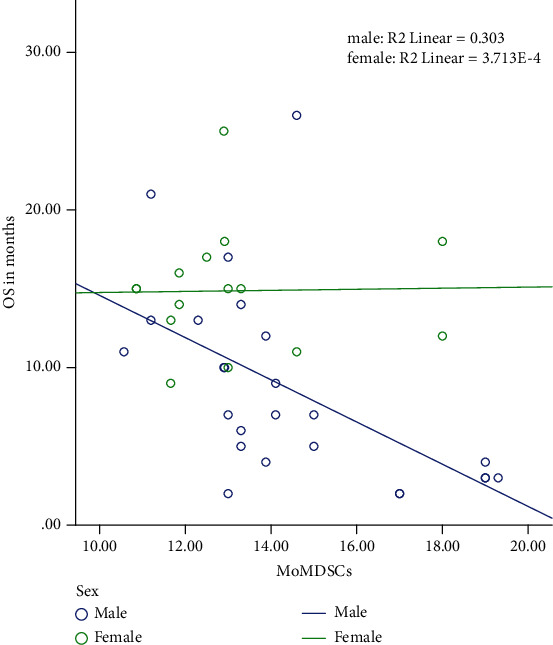
Correlation between overall survival (OS) and myeloid-derived suppressor cells (MDSCs) according to sex.

The mean Mo-MDSC percentage expressed negative correlations with OS, MPV/PC ratio, LMR, and with the number of cycles of chemotherapy received (Table 4); further analysis demonstrated that a negative correlation between OS and Mo-MDSCs was clearly apparent in males but not in females (Figure 2).

**Figure 3:**
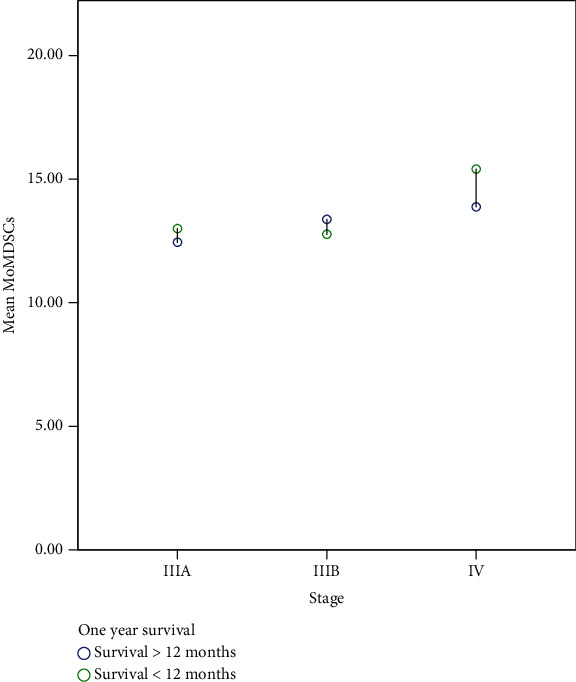
Differences in the mean myeloid-derived suppressor cells (Mo-MDSCs) according to 1-year survival in each stage, *p* < 0.0001.

Eight out of nine, 10/14, and 1/17 patients with stages IIIA, IIIB, and IV, respectively, achieved more than one-year OS compared to 1/9, 4/14, and 16/17 patients of the previous stages that had lower than one-year OS and the results were significant (*p* < 0.0001); additionally, the mean percentage of Mo-MDSCs for those with more than one-year survival was 13.01 compared with 14.79 for those with lower than one-year survival (*p* = 0.021, Figure 3).

### 3.3. Overall Survival

**Figure 4:**
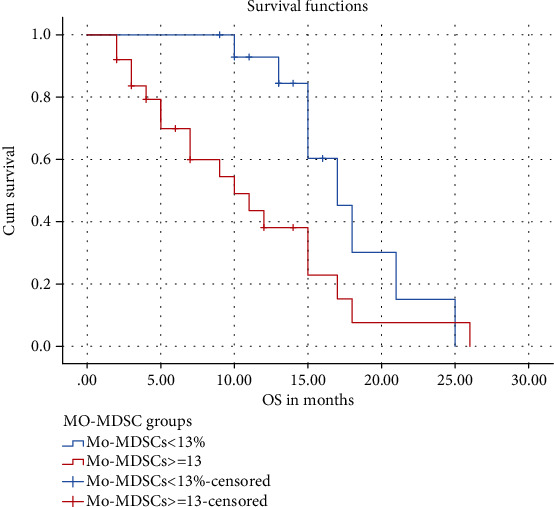
Differences in overall survival (OS) according to myeloid-derived suppressor cell (Mo-MDSC) cutoff value.

The ROC curve was performed to find a cutoff point of Mo-MDSCs % at which the overall survival significantly differed, and it was ≈13% with AUC = 0.742 ± 0.1 (95%CI = 0.59–0.9, *p* = 0.009); OS was significantly higher for those with Mo‐MDSCs < 13% than those with Mo‐MDSCs ≥ 13% (17.64 vs. 11.0, *p* = 0.022) (Table 5, Figure 4).

**Figure 5:**
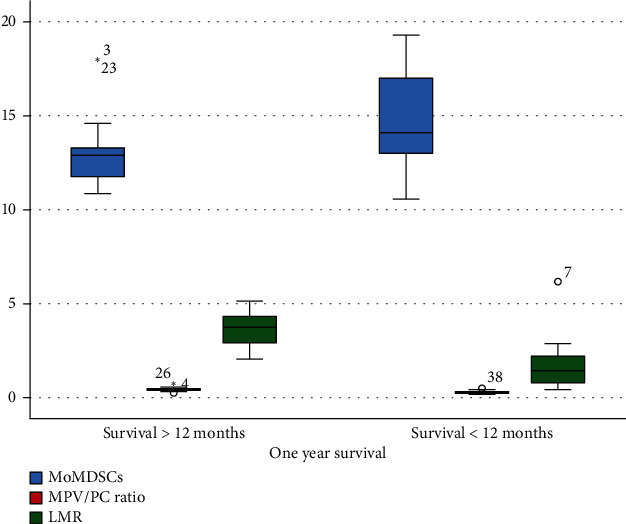
Differences in myeloid-derived suppressor cells (Mo-MDSCs), lymphocyte to monocyte ratio (LMR), and mean platelet volume to platelet count (MPV/PC) ratio for patients with >12 months of survival compared to patients <12 months of survival, data analyzed by Mann–Whitney test and independent sample *t*-test.

The mean ± SD for LMR and MPV/PC ratio for patients with >12 months of survival were 3.63 ± 0.92 and 0.44 ± 0.1 compared to 1.7 ± 1.3 and 0.29 ± 0.1 for those with <12 months of survival (*p* < 0.0001 and *p* < 0.0001, respectively) (Figure 5).

**Figure 6:**
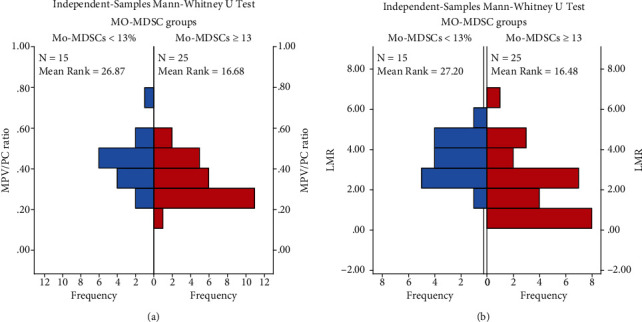
(a) Differences of MPV/PC according to cutoff point values of Mo-MDSCs. (b) Differences of LMR according to cutoff point values of Mo-MDSCs.

Furthermore, the distribution of MPV/PC and LMR was significantly different according to the cutoff point value of Mo-MDSCs, where they significantly accumulated in those with Mo‐MDSCs < 13% compared to those with ≥13% (for MPC/PV, 0.4 ± 0.1 vs. 0.3 ± 0.1, *p* =0.008; for LMR, 3.4 ± 1.11 vs. 2.2 ± 1.5, *p* = 0.005) (Figures 6(a) and 6(b)).

### 3.4. Multiple Linear Regression Test for Different Predictors of OS

Multiple linear regression was run to predict OS from 8 predictors found to significantly affect the mean OS including age, sex, smoking, stage, performance status, Mo-MDSCs, MPV/PC ratio, and LMR; these prementioned variables collectively predicted OS with significant impact (*F*(8, 31) = 14.230, *p* < 0.0001, *R*^2^ = 0.786); however, only stage and LMR added significantly to the prediction of OS. Looking at Mo-MDSCs, *B* = 0.018 and *p* = 0.9 denoted that only 1.8% of the change in OS variance was attributed to Mo-MDSC change when all remaining predictors were held constant. Furthermore, it was misleading as for each one of the percentage increase in Mo-MDSCs, there was an increase in OS by 0.018 months; in addition, it was not significant (Table 6).

## 4. Discussion

Nowadays, it is well established that the tumor microenvironment and immune system play a crucial role in the initiation and progression of different cancers, including NSCLC [22]. MDSCs are considered the major suppressor of the immune system interfering with both innate and adaptive immune responses. Mo-MDSCs were detected to be highly expressed in peripheral blood than excised tissues and lymph nodes of NSCLC patients [23]; subsequently, flow cytometric analysis of peripheral blood for detection of these cells is a reliable method.

In the current study, Mo-MDSCs were significantly more prevalent in the peripheral blood of NSCLC patients than healthy controls. Furthermore, increased levels of these cells were associated with poor prognostic features, including advanced stage, low LMR, low MPV/PC ratio, and poor overall survival.

Mo-MSDCs were reported to produce TGF-*β* in the peripheral blood [24]; furthermore, TGF-*β* was known to induce immunosuppression and promote angiogenesis in TME; in another study, this cytokine was produced by Mo-MDSCs in all tissues [23].

Several studies demonstrated that NSCLC was associated with high levels of Mo-MDSCs which in turn were responsible for the resistance of this tumor to different systemic therapies [21, 25–27].

Yamauchi et al. [28] showed that a significant increase in the percentage of circulating Mo-MDSCs was observed in patients with resectable non-small-cell lung cancer compared with healthy donors; in addition, preoperative levels of Mo-MDSCs predicted recurrence-free survival after surgery.

A meta-analysis of 40 studies supported the existence of an association between higher MDSC levels and worse clinical outcomes in solid malignancies including NSCLC where total MDSCs (HR = 3.386; 95%CI = 1.618–7.087), M-MDSCs (HR = 2.834; 95%CI = 2.128–3.774), and PMN-MDSCs (HR = 1.915; 95%CI = 1.420–2.583) were all associated with worse disease-free survival, progression-free survival, and recurrence-free survival [29].

In accordance with the previously mentioned studies, our results depicted significantly increased levels of Mo-MDSCs in patients compared with healthy controls in addition to an association of these cells with poor survival in NSCLC patients.

Interestingly, immunophenotyping analysis was performed on peripheral blood samples from seven patients with lung cancer unfit for surgery and treated with stereotactic body radiotherapy (SBRT) to evaluate the impact of SBRT on patients' immune cells including Mo-MDSCs and reported a significant decrease in these cells after RT [30] implicating that not only chemotherapy affected the levels of immune system; however, there was no uniform effect of chemotherapy on the peripheral blood percentages or immunosuppressive function of Mo-MDSCs, but three cycles of bevacizumab-based chemotherapy were associated with significantly reduced level of these cells [31]. Our study agreed with the previous one, where the percentage of Mo-MDSCs negatively correlated with the number of cycles of chemotherapy.

The percentage of MDSCs in patients with colorectal cancer with LMR ≤ 2.4 was statistically higher than that with LMR > 2.4 (*p* = 0.012). Those patients with LMR ≤ 2.4 exhibited a statistically lower RFS than those with LMR > 2.4 (*p* = 0.008) [32]. Likewise, a negative correlation between LMR and Mo-MDSCs was evident in our work with significant effect (*r* = −0.446, *p* = 0.004).

Wang et al. proved that low pretreatment LMR was with poor OS (HR = 1.63, 95%CI : 1.44–1.85, *p* < 0.001) and PFS (HR = 1.49, 95%CI : 1.25–1.77, *p* < 0.001) compared to those NSCLC patients with high LMR in their meta-analysis of 20 articles discussing the role of LMR in NSCLC [33].

MPV/PC was established as a prognostic factor in both univariate and multivariate analysis of NSCLC with a cutoff value of 0.408730, where low MPV/PC ratio was associated with poor OS (hazard ratio (HR): 1.668, 95%CI : 1.235–2.271, *p* = 0.0008) [34]; also, low MPV/PC ratio was associated with poor prognostic features including advanced stage and poor performance status in these patients [35]. Our results in turn adhered to the previous studies where LMR and MPV/PC ratio were negatively correlated with OS.

The inverse relation between MDSCs and LMR remains to be elucidated; increased peripheral MDSCs contribute to peripheral monocytosis and hence lower LMR; in addition, it is established that peripheral monocytosis has been reported to be associated with poor clinical outcomes [32]; we reported significant monocytosis in NSCLC patients compared to their controls with negative correlation between LMR and Mo-MDSCs in our patients.

It is worth mentioning that limited data are available to adequately enforce our finding regarding the negative correlation between Mo-MDSCs and MPV/PC ratio; however, in line with previous studies relating MPV/PC ratio to poor prognosis in NSCLC, subsequently, a low MPV/PC ratio may be correlated with increased Mo-MDSCs.

Several limitations exist in the current study, including the small number of enrolled patients, immunohistochemical evaluation of MDSCs in tumor tissues not done, and heterogeneity of the studied patients. Future work is recommended including addition of CD14, CD11b, CD15, and CD66b immunohistochemistry and qPCR of tissue to verify the hypothesis further. Also, data about the proinflammatory cytokines including IL6, IL8, and TNF*α* are needed to demonstrate the relationship between inflammation factors and MDSCs.

In conclusion, Mo-MDSCs represent one of the key mechanisms in immunosuppressive TME to play major roles not only in the carcinogenesis of lung cancer but also in disease progression and prognosis and, in addition, predict the efficacy of immune checkpoint inhibitors; our results provided some support to target Mo-MDSCs and needed to be augmented by further studies.

## Figures and Tables

**Table 1:** Differential accumulation of Mo-MDSCs between NSCLC patients and healthy controls.

	NSCLC (mean ± SE)	Controls (mean ± SE)	*p* value
Mo-MDSCs	13.99 ± 0.55	4.15 ± 0.2	<0.0001
Absolute monocytic count	12.29 ± 0.32	7.41 ± 0.29	<0.0001

Data expressed as mean ± SE and analyzed by Mann–Whitney *U* test.

**Table 2:** Clinicopathologic characteristics of 40 patients with NSCLC.

Characteristics	Descriptive
Age (mean ± SE)	58.5 ± 1.7
Median	59.5 y
Sex (male/female)	25/15
Smoking	
Never smoker	17 (42.5%)
Current smoker	11 (27.5%)
Past smoker	12 (30%)
ECOG-PS	
PS = 1	12 (30%)
PS = 2	13 (32.5%)
PS = 3	15 (37.5%)
Histopathology	
Squamous cell carcinoma	12 (30%)
Adenocarcinoma	23 (57.5%)
Large cell carcinoma	4 (10%)
Bronchoalveolar carcinoma	1 (2.5%)
Stage	
IIIA	9 (22.5%)
IIIB	14 (35%)
IV	17 (42.5%)
Median number of chemotherapy cycles	3 (range: 0–7)
LMR (mean ± SE)	2.62 ± 0.23
MPV/PC ratio (mean ± SE)	0.36 ± 0.02
Relative monocytic count	12.29 ± 0.22
Relative lymphocytic count	20.0 ± 1.94
Outcome	
Dead	26 (65%)
Alive	14 (35%)

LMR: lymphocyte to monocyte ratio; MPV/PC ratio: mean platelet volume to platelet count ratio; ECOG-PS: Eastern Cooperative Oncology Group-performance status.

**Table 3:** Relation of Mo-MDSCs to different clinical characteristics.

Characteristics	Mo-MDSCs (mean ± SE)	*p* value
Sex		0.1
Male	14.43 ± 0.51	
Female	13.13 ± 0.57	
Smoking		0.2
Never smoker	13.13 ± 0.5	
Current smoker	14.93 ± 0.72	
Past smoker	14.2 ± 0.83	
ECOG-PS		0.09
PS = 1	13.38 ± 0.54	
PS = 2	13.21 ± 0.73	
PS = 3	15.04 ± 0.66	
Histopathology		0.8
Squamous cell carcinoma	13.74 ± 0.83	
Adenocarcinoma	14.0 ± 0.49	
Large cell carcinoma	13.0 ± 0.0	
Bronchoalveolar carcinoma	15.2 ± 1.3	
Stage		0.0.003^∗^
IIIA	12.51 ± 0.4	
IIIB	13.2 ± 0.61	
IV	15.32 ± 0.61	

Data analyzed by Mann–Whitney *U* test and Kruskal-Wallis test. *r*: Spearman coefficient; ^∗^significant.

**Table 4:** Correlation between Mo-MDSCs and survival.

		Mo-MDSCs	Age	MPV/PC ratio	Number of cycles	OS in months	LMR
Mo-MDSCs	*r* *p*	—	0.016	-0.464	-0.412	-0.471	-0.446
		—	0.920	0.003	0.008	0.002	0.004
Age	*r* *p*	0.016	—	-0.215	0.014	-0.248	-0.209
		0.920		0.183	0.931	0.123	0.195
MPV/PC ratio	*r* *p*	-0.464	-0.215	—	-0.010	0.737	0.548
		0.003	0.183	—	0.951	<0.0001	<0.0001
Number of cycles	*r* *p*	-0.412	0.014	-0.010	—	0.036	0.285
		0.008	0.931	0.951	—	0.826	0.075
OS in months	*r* *p*	-0.471	-0.248	0.737	0.036	—	0.718
		0.002	0.123	0.0001	0.826	—	<0.0001
LMR	*r* *p*	-0.446	-0.209	0.548	0.285	0.718	—
		0.004	0.195	0.0001	0.075	<0.0001	—

Data analyzed by Spearman rho correlation. *r*: correlation coefficient.

**Table 5:** Difference of OS according to Mo-MDSC levels among NSCLC patients.

OS	Mo‐MDSCs < 13%	Mo‐MDSCs ≥ 13%
Mean ± SE	17.64 ± 1.51	11.0 ± 1.6
95% CI	14.7-20.6	7.9-14.04
Logrank, *p* value	5.21, *p* = 0.022	

Data analyzed by Kaplan-Meier with logrank for comparison.

**Table 6:** Multiple linear regression test of different predictors of OS.

	Unstandardized coefficients		*t*	Sig.	95% confidence interval for *B*	
	*B*	Std. error			Lower bound	Upper bound
(Constant)	6.817	7.540	0.904	0.373	-8.561	22.196
Mo-MDSCs	0.018	0.252	0.073	0.943	-0.496	0.533
Smoking	0.804	1.102	0.730	0.471	-1.444	3.052
MPV/PC ratio	12.246	6.465	1.894	0.068	-0.939	25.431
ECOG-PS	-1.141	0.887	-1.286	0.208	-2.950	0.668
Sex	1.739	2.046	0.850	0.402	-2.435	5.913
Stage	-2.926	1.033	-2.832	0.008	-5.033	-0.819
LMR	1.295	0.506	2.561	0.016	0.264	2.326
Age	0.017	0.063	0.279	0.782	-0.111	0.146

Data analyzed by linear regression with enter method.

## Data Availability

The data used to support the findings of this study are available from the corresponding authors upon request.
